# SMYD2 aggravates gastrointestinal stromal tumor via upregulation of EZH2 and downregulation of TET1

**DOI:** 10.1038/s41420-022-01038-w

**Published:** 2022-06-06

**Authors:** Yong Ji, Xiaofeng Xu, Cong Long, Jianjiang Wang, Li Ding, Zhizhong Zheng, Huiping Wu, Liu Yang, Lan Tao, Feng Gao

**Affiliations:** 1 Department of General Gastrointestinal Surgery, Jingjiang People’s Hospital, 214500 Jingjiang, P.R. China; 2 Department of Clinical Laboratory, Jingjiang People’s Hospital, 214500 Jingjiang, P.R. China; 3 Department of General Surgery, Jingjiang People’s Hospital, 214500 Jingjiang, P.R. China; 4 Department of Science and Education, Jingjiang People’s Hospital, 214500 Jingjiang, P.R. China; 5 Central Laboratory, Jingjiang People’s Hospital, 214500 Jingjiang, P.R. China

**Keywords:** Cancer, Diseases

## Abstract

SMYD2, as an oncogene, has been involved in multiple types of cancer, but the potential role of SMYD2 in gastrointestinal stromal tumors (GIST) remains enigmatic and requires further investigation. Hence, this study was conducted with the main objective of analyzing the effect of SMYD2 on GIST. GIST and adjacent normal tissues were collected from 46 patients with GIST where the expression of EZH2, SMYD2, and TET1 was determined, followed by the analysis of their interactions. The functional role of SMYD2 in cell biological functions was determined using a loss-of-function assay in GIST-T1 cells. Nude mouse xenograft experiments were performed to verify the role of the SMYD2/EZH2/TET1 axis in GIST in vivo. EZH2 was upregulated in GIST tissues and cell lines, which was positively correlated with SMYD2 expression and inversely correlated with TET1 expression in GIST tissues. EZH2 silencing due to SMYD2 inhibition reduced GIST-T1 cell proliferation and accelerated cell senescence. EZH2 repressed TET1 expression by promoting H3K27me3 methylation in the TET1 promoter region. TET1 inhibition reversed the effect of EZH2 silencing on the biological functions of GIST-T1 cells. In vivo data further revealed the promoting effect of SMYD2 on the progression of GIST by regulating the EZH2/TET1 axis. Overall, this study demonstrates that SMYD2 can increase EZH2 expression while suppressing TET1 expression, thus accelerating GIST, and creating new treatment opportunities for GIST.

## Introduction

Gastrointestinal stromal tumor (GIST), often characterized by carcinomatous changes in the stomach or the small intestine, is the most common mesenchymal tumor of the alimentary tract [1]. Up to 20% GIST patients present with remarkable metastasis at diagnosis, with this cancer, typically metastasizing to the liver, and/or throughout the serosal surfaces of the abdomen [2]. Due to the high resistance to conventional chemotherapy and radiotherapy, patients with GIST have a poor prognosis [1]. In addition, the genetically and biologically heterogeneous nature of GIST was correlated with epigenetic regulation [3]. For instance, the genetic hallmarks of GIST patients have been revealed to show constitutively activating mutations of proto-oncogene *KIT* (~85% of cases) and *PDGFRA* (~7% of cases) which encodes platelet-derived growth factor receptor alpha (PDGFRα) [4]. With these findings serving as the basis, we conducted the present study to shed a light on the molecular mechanism underlying pathogenesis.

Post-translational modification, especially methylation of histone or non-histone proteins, has been observed to play a crucial role in tumorigenesis in numerous cancers [5]. Enhancer of zeste homolog 2 (EZH2), one of the histone methyl transferases, is the key transcriptional regulator involved in histone H3 lysine 27 trimethylation [6]. There are extensive data demonstrating the essential role of EZH2 in the incidence of multiple human malignancies such as multiple myeloma, lymphoma, melanoma, thyroid, prostate, breast, bladder, and liver cancers [7–9]. However, the role of EZH2 in GIST development remains unclear. Importantly, a prior study elucidated that SET and MYND domains containing 2 (SMYD2) can stabilize EZH2 via direct methylation at the K307 site of EZH2 [10]. Interestingly, there are numerous studies elaborating on the association between SMYD2 and a range of tumors [11–13]. Furthermore, it was noted from prior research that SMYD2 possessed oncogenic effects on gastric cancer by accelerating cell proliferation [14]. In addition, EZH2 resulted in the downregulation of TET1 by H3K27me3 epigenetic regulation in triple-negative breast cancer (TNBC), subsequently diminishing p53 expression [15]. Notably, TET1 upregulation was observed to result in limited cell invasion and migration in gastric cancer [16]. TET1 functions as a DNA demethylase that regulates gene expression by altering the statue of DNA methylation [17]. Fu et al. revealed that in gastric cancer cells, TET1 participated in DNA-PK activation of p53 via DNA demethylation [18]. Moreover, the correlation between p53 and GIST prognosis has been observed in a prior study [19]. Thus we hypothesized that SMYD2-mediated EZH2 methylation was essential in the pathogenesis of GIST by regulating the TET1/p53 axis and conducted tissue, cell, and animal experiments to validate this hypothesis.

## Results

### EZH2 silencing limited proliferation and induced senescence of GIST cells

**Fig. 1:**
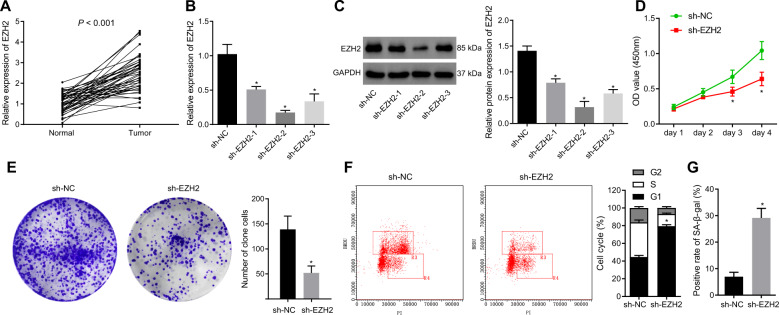
EZH2 silencing inhibits the proliferation and induces the senescence of GIST cells. **A** RT-qPCR detection of EZH2 expression in cancer and adjacent normal tissues from 46 GIST patients. **B** RT-qPCR detection of EZH2 expression in GIST-T1 cells treated with sh-EZH2-1, sh-EZH2-2, or sh-EZH2-3. **C** Western blot analysis of the expression of EZH2 in GIST-T1 cells treated with sh-EZH2-1, sh-EZH2-2, or sh-EZH2-3. **D** CCK-8 detection of GIST-T1 cell viability after silencing EZH2. **E** GIST-T1 cell colony formation ability after silencing EZH2 evaluated by soft agar colony formation assay. **F** BrdU/PI cell cycle analysis of GIST-T1 cell cycle distribution after silencing EZH2. **G** SA-β-gal detection of ratio of senescent GIST-T1 cells after silencing EZH2 (1 mL SA-β-gal is used per experiment). *p < 0.05 vs. the adjacent normal tissues or sh-NC-treated GIST-T1 cells. Measurement data were expressed as mean ± standard deviation. Comparison between cancer tissues and adjacent normal tissues was conducted using paired *t*-test, while comparison between the other two groups was conducted by unpaired *t*-test. The cell experiment was repeated three times.

Multiple studies have shown the association of the mutation and overexpression of EZH2 with the development of several types of cancer; for instance, EZH2 overexpression assumes an essential role in anaplastic thyroid cancer [8]. High expression of EZH2 relates to the poor prognosis of multiple myeloma [9]. Currently, the function of EZH2 in GIST remained unclear. To investigate the role of EZH2 in GIST, we harvested cancer tissues and adjacent normal tissues from 46 GIST patients, and the results of RT-qPCR showed markedly increased EZH2 expression in GIST tissues (Fig. 1A). Meanwhile, EZH2 expression was increased in human GIST cell lines GIST-T1, GIST-48, and GIST-882 compared with GES-1 cell line, of which GIST-T1 cell line had the highest expression of EZH2 (Fig. S1) and was thus selected for subsequent experiments. As reflected by RT-qPCR and Western blot analysis, EZH2 expression was decreased in GIST-T1 cells treated with sh-EZH2-1, sh-EZH2-2, or sh-EZH2-3, with the lowest expression observed in GIST-T1 cells treated with sh-EZH2-2 (Fig. 1B, C). Therefore, the following experiments were implemented with sh-EZH2-2. As shown in Fig. 1D, E, we depicted limited viability and colony formation of EZH2-silenced GIST-T1 cells. In addition, BrdU/PI cell cycle analysis displayed that EZH2-silenced GIST-T1 cells were significantly blocked in the G1 phase (Fig. 1F). Moreover, the results of SA-β-gal staining revealed that the number of senescent GIST-T1 cells was potently elevated by silencing EZH2 (Fig. 1G). Therefore, silencing of EZH2 contributed to suppressed GIST cell proliferation and promoted cell senescence.

### SMYD2 inhibition weakened the proliferation and facilitated the senescence of GIST cells by decreasing the expression of EZH2

**Fig. 2:**
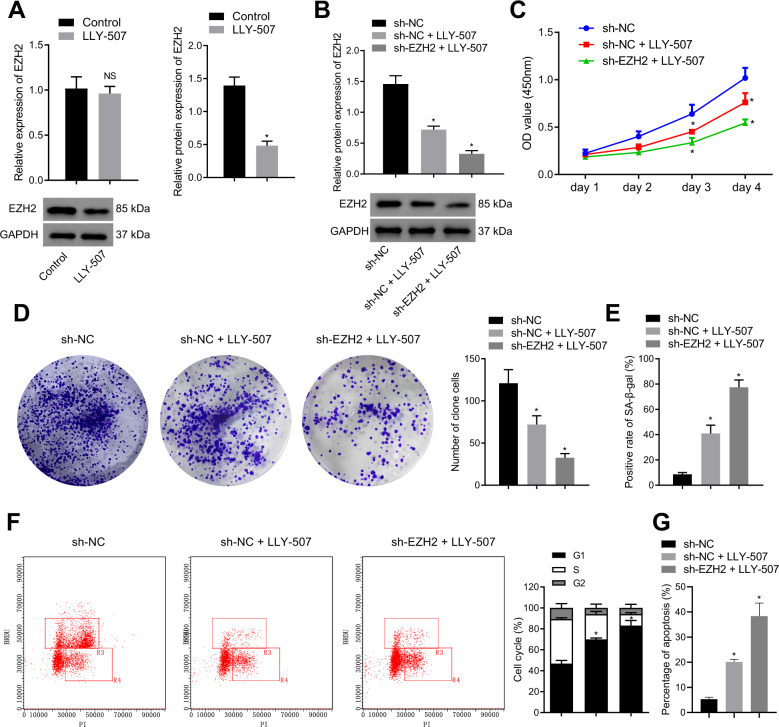
SMYD2 inhibition represses the proliferation and accelerates the senescence of GIST cells through reducing EZH2 expression. **A** RT-qPCR and western blot analysis of EZH2 expression in GIST-T1 cells treated with LLY-507 (20 nM). GIST-T1 cells were treated with LLY-507 or combined with sh-EZH2. **B** Western blot analysis of EZH2 protein expression in GIST-T1 cells. **C** CCK-8 assay of GIST-T1 cell viability. **D** GIST-T1 cell colony formation ability evaluated by soft agar colony formation assay. **E** SA-β-gal detection of the proportion of senescent GIST-T1 cells. **F** GIST-T1 cell cycle distribution measured by BrdU/PI cell cycle assay. **G** Annexin V-FITC/PI double staining of GIST-T1 cell apoptosis. *p < 0.05 *vs*. control or sh-NC- or sh-NC + LLY-507-treated GIST-T1 cells. Measurement data were expressed as mean ± standard deviation. Unpaired *t*-test was used for two-group data comparison. The cell experiment was repeated three times.

Histone methyltransferase SMYD2 has been reported to directly methylate at the K307 of EZH2, thereby stabilizing EZH2 [10]. SMYD2 overexpression is associated with the proliferation and poor prognosis of human non-papillomavirus-related head and neck cancer tumor cells [11], cell proliferation of esophagus and squamous cell carcinoma [20], and the incidence of cervical cancer [21]. Several inhibitors for SMYD2 have been reported for the beneficial effects of suppressing cancer. Here, we selected the small molecule inhibitors of SMYD2, LLY-507, and AZ-505, which could specifically inhibit the methyltransferase activity of SMYD2 [22, 23]. As reported, SMYD2 promotes the stability of EZH2 in breast cancer cells and does not affect the transcription of EZH2 [10], Based on the aforementioned information, we studied the role of SMYD2 inhibitors LLY-507 and AZ-505 in the transcription and translation of EZH2 in GIST-T1 cells. LLY-507 (20 nM) or AZ-505 (20 nM) was added to GIST-T1 cells. It was described that after inhibiting SMYD2, EZH2 mRNA expression did not change but the protein expression was strikingly reduced (Figs. 2A, S2-A). Therefore, LLY-507 (20 nM) or AZ-505 (20 nM) was supplemented to sh-NC- or sh-EZH2-treated GIST-T1 cells. Western blot analysis results manifested that compared with sh-NC-treated GIST-T1 cells, EZH2 protein expression was remarkably diminished in GIST-T1 cells treated with sh-NC + LLY-507, sh-EZH2 + LLY-507, sh-NC + AZ-505, or sh-EZH2 + AZ-505. Moreover, EZH2 protein expression was lower in GIST-T1 cells treated with sh-EZH2 + LLY-507 or sh-EZH2 + AZ-505 than in GIST-T1 cells treated with sh-NC + LLY-507 or sh-NC + AZ-505 (Figs. 2B, S2-B).

Furthermore, the half-life of EZH2 was shortened in cells overexpressing FLAG EZH2 K307R following treatment with CHX and MG132 (Fig. S3-A). This indicated that SMYD2 methylated EZH2 at K307 and could maintain EZH2 stability through proteasome-mediated degradation. In addition, the demethylation level of K307 and the abundance of EZH2 were significantly reduced after LLY-507 treatment (Fig. S3-B). After treatment with LLY-507, the methylation level of p53 K310 was also reduced (Fig. S3-C). Therefore, SMYD2 promoted the stability of EZH2 through the methylation modification at K307.

As documented in Figs. 2C, D, S2-C, D, experimental results revealed that GIST-T1 cell viability and colony formation ability were declined by treatment with sh-NC + LLY-507, sh-EZH2 + LLY-507, sh-NC + AZ-505, or sh-EZH2 + AZ-505, especially treatment with sh-EZH2 + LLY-507 or sh-EZH2 + AZ-505. Besides, SA-β-gal staining and BrdU/PI cell cycle analysis data demonstrated that compared with sh-NC treatment, GIST-T1 cell senescence was augmented and G1 phase-arrested cells were increased after treatment with sh-NC + LLY-507, sh-EZH2 + LLY-507, sh-NC + AZ-505, or sh-EZH2 + AZ-505. In addition, sh-EZH2 + LLY-507 or sh-EZH2 + AZ-505 treatment led to enhancement of GIST-T1 cell senescence and G1 phase-arrested cells in contrast to sh-NC + LLY-507 or sh-NC + AZ-505 treatment (Figs. 2E, F, S2-E, F). Additionally, the results of Annexin V-FITC/PI double staining revealed an increase in the cell apoptosis upon treatment with sh-NC + LLY-507, sh-EZH2 + LLY-507, sh-NC + AZ-505, or sh-EZH2 + AZ-505, with more obvious cell apoptosis noted in the presence of sh-EZH2 + LLY-507 and sh-EZH2 + AZ-505 (Figs. 2G, S2-G). Additionally, we repeated all the above-mentioned experiments in GIST-T1 cells with SMYD2 silencing and the results obtained were consistent with those upon treatment with SMYD2 inhibitors (Fig. S4-A-G). The above results suggested that SMYD2 inhibition participated in the suppression of GIST cell proliferation and induction of cell senescence by downregulating EZH2 expression.

### EZH2 diminished TET1 expression via promotion of H3K27me3 methylation of TET1

**Fig. 3:**
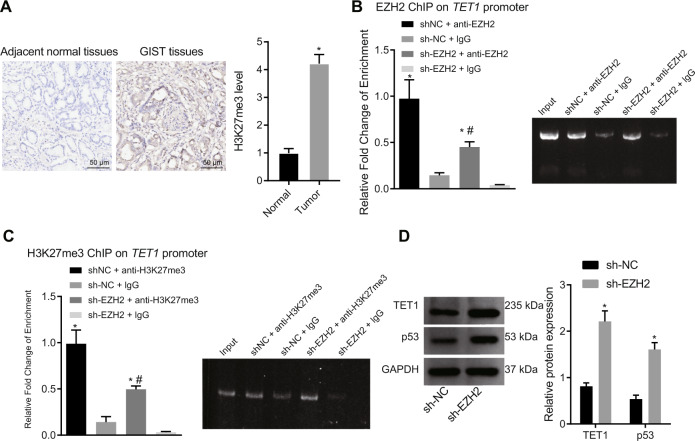
EZH2 decreases TET1 expression via enhancement of H3K27me3 methylation in the promoter region of TET1. **A** H3K27me3 level in GIST tissues and adjacent normal tissues measured by immunohistochemistry, scale bar: 50 μm. **B** qPCR results of the TET1 promoter region (EZH2 antibody) analyzed by ChIP (left) and the agarose gel electrophoresis detection results (right). **C** qPCR results of the TET1 promoter region (H3K27me3 antibody) analyzed by ChIP (left) and the agarose gel electrophoresis detection results (right). **D** Western blot analysis of TET1 and p53 proteins in sh-EZH2-treated GIST-T1 cells. *p < 0.05 vs. adjacent normal tissues, sh-NC-treated GIST-T1 cells or GIST-T1 cells incubated with sh-NC + IgG. ^#^*p* < 0.05 vs. GIST-T1 cells incubated with sh-EZH2 + IgG. Measurement data were expressed as mean ± standard deviation. Unpaired *t*-test was used for two-group data comparison. The cell experiment was repeated three times.

In TNBC, EZH2 can epigenetically modify TET1 through H3K27me3, thereby inhibiting the expression of the tumor suppressor gene TET1 (an activator of p53 tumor suppressor signaling pathway). Thus, EZH2 can promote tumorigenesis by inhibiting the TET1/p53 signaling [15]. Besides, evidence has shown that TET1 is downregulated in gastric cancer caused by H. pylori infection [24]. Here, we aimed to examine whether EZH2 participated in GIST by acting on TET1. Immunohistochemistry revealed an elevation in H3K27me3 level in the GIST tissues (Fig. 3A). ChIP data indicated that the enrichment of EZH2 was decreased in the TET1 promoter region in cells treated with sh-EZH2 (Fig. 3B). In addition, in the presence of sh-EZH2, the enrichment of H3K27me3 was reduced in the TET1 promoter region (Fig. 3C). ChIP assay was used to analyze the enrichment of TET1 in the TP53 promoter region and RT-qPCR results showed that the level of TET1 in the TP53 promoter region was significantly reduced by sh-EZH2 (Fig. S5-A). Furthermore, TET1 and p53 protein expression was prominently increased in the EZH2-silenced GIST-T1 cells (Fig. 3D). Meanwhile, the expression of p53, p53-S15, and p21 was downregulated in response to sh-TET1 treatment (Fig. S5-B). In summary, EZH2 suppressed TET1 in GIST cells by promoting H3K27me3 methylation in the TET1 promoter region.

### TET1 silencing reversed the effect of EZH2 silencing on GIST cells

**Fig. 4:**
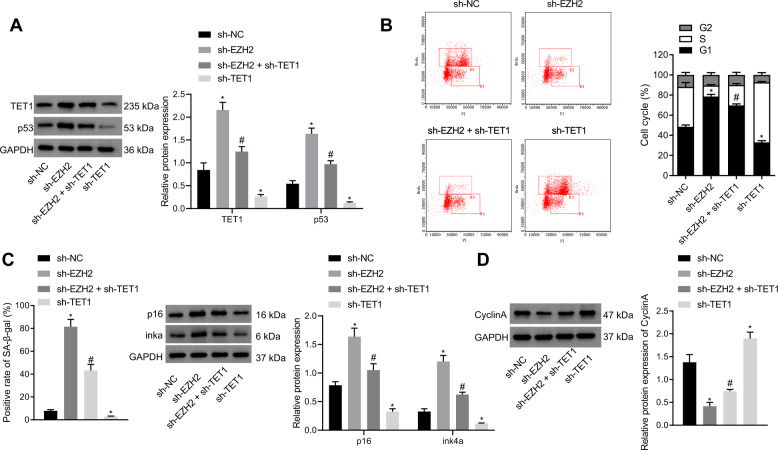
TET1 silencing abolishes the effect of EZH2 silencing on GIST cells. GIST-T1 cells were treated with sh-NC, sh-EZH2, sh-TET1, or sh-EZH2 + sh-TET1. **A** Western blot analysis of TET1 and p53 protein expression in GIST-T1 cells. **B** BrdU/PI cell cycle assay to quantify GIST-T1 cell cycle distribution. **C** SA-β-gal detection of ratio of senescent GIST-T1 cells. **D** Western blot analysis of cyclin A protein expression in GIST-T1 cells. *p < 0.05 vs. sh-NC-treated GIST-T1 cells, ^#^*p* < 0.05 vs. sh-EZH2-treated GIST-T1 cells. Measurement data were expressed as mean ± standard deviation. Unpaired *t*-test was used for two-group data comparison. One-way ANOVA with Tukey’s post-hoc test was used to compare multigroup data. The cell experiment was repeated three times.

After verifying the relationship between TET1 and EZH2 in GIST, we focused on examining the effect of EZH2 on the biological function of GIST cells by inhibiting the expression of TET1. Western blot analysis results exhibited that the expression of TET1 and p53 was upregulated in sh-EZH2-treated GIST-T1 cells, which was negated by sh-TET1 treatment (Fig. 4A). The findings from BrdU/PI cell cycle analysis revealed more G1 phase-arrested GIST-T1 cells after EZH2 silencing, which was counteracted by silencing of TET1 (Fig. 4B). Consistently, SA-β-gal staining results manifested that the proportion of senescent GIST-T1 cells after silencing EZH2 was severely enhanced, which was annulled by sh-TET1 treatment (Fig. 4C). Additionally, a reduction in the cyclin A expression was seen in sh-EZH2-treated GIST-T1 cells, which was abrogated by sh-TET1 (Fig. 4D). Conclusively, EZH2 silencing upregulated the expression of TET1 and p53 to block the cell cycle and promote cell senescence. In contrast, silencing of TET1 could reverse the effect of silencing EZH2 on GIST-T1 cells.

### EZH2 expression was positively correlated with SMYD2 expression and inversely correlated with TET1 expression in GIST tissues

**Fig. 5:**
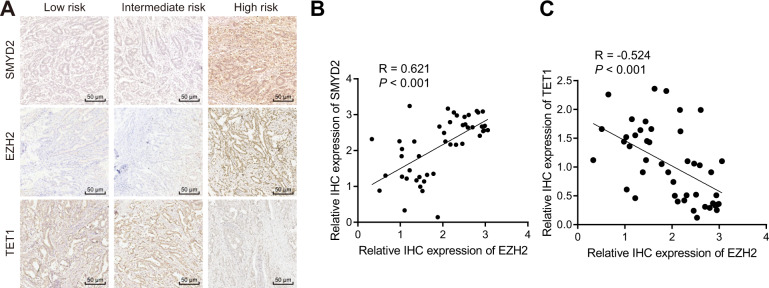
Correlation analysis among SMYD2, EZH2, and TET1 expression in GIST tissues. **A** SMYD2, EZH2, and TET1 expression in the tumor tissues collected from GIST patients with low risk, intermediate risk, and high risk measured by immunohistochemistry, scale bar: 50 μm. **B** Correlation of the SMYD2 expression with EZH2 expression in the GIST tissue analyzed by Pearson’s correlation coefficient. **C** Correlation of EZH2 expression and TET1 expression in the GIST tissue analyzed by Pearson’s correlation coefficient. Measurement data were expressed as mean ± standard deviation.

To verify the specific relationship between SMYD2, EZH2, and TET1 in GIST, the expression of SMYD2, EZH2, and TET1 was first detected in the tumor tissues collected from GIST patients with low risk, intermediate risk, and high risk using immunohistochemistry (Fig. 5A). After quantification of the positive signals, Pearson’s correlation coefficient documented that SMYD2 expression and EZH2 expression shared a positive correlation (Fig. 5B), while TET1 expression and EZH2 expression presented a negative correlation in GIST tissues (Fig. 5C). The results demonstrate correlations among SMYD2, EZH2s, and TET1 expression in the tumor tissues of GIST patients, further verifying the results of the previous experiments from a clinical perspective.

### SMYD2 accelerated the progression of GIST in vivo by regulating the EZH2/TET1 axis

**Fig. 6:**
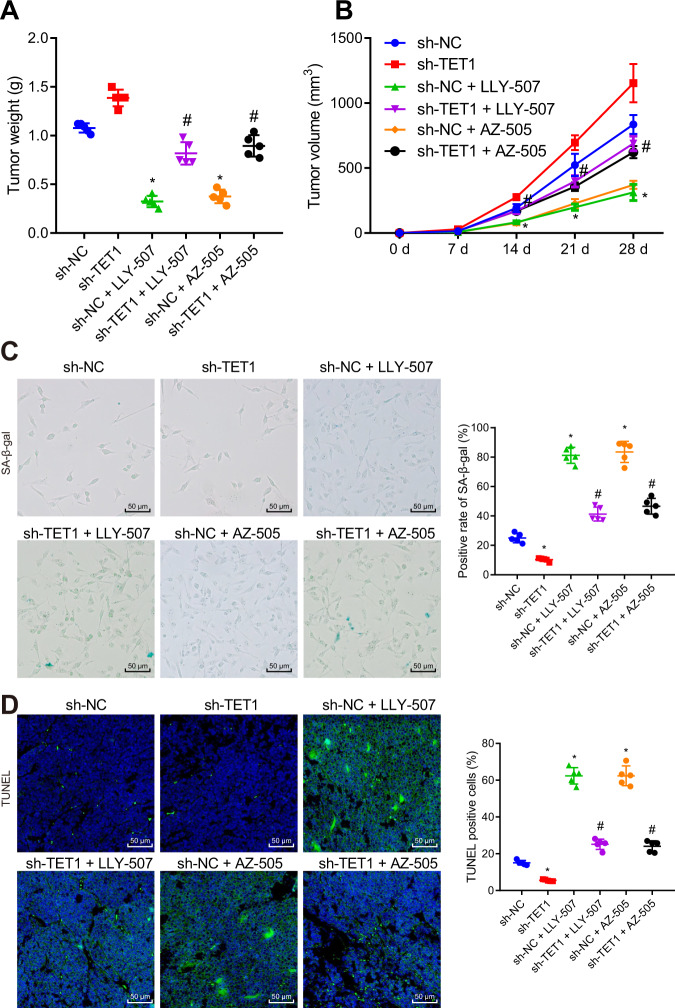
SMYD2 promotes the progression of GIST in vivo by mediating the EZH2/TET1 axis. The nude mice were treated with sh-NC, sh-TET1, sh-NC + LLY-507, sh-TET1 + LLY-507, sh-NC + AZ-505, or sh-TET1 + AZ-505. **A** Weight of tumors. **B** Volume of tumors. **C** SA-β-gal detection of cell senescent in tumor tissues, scale bar: 50 μm. **D** TUNEL detection of apoptotic cells in the tumor tissues, scale bar: 50 μm. *p < 0.05 *vs*. sh-NC-treated mice, ^#^*p* < 0.05 vs. mice treated with sh-NC + LLY-507 or sh-NC + AZ-505. Measurement data were expressed as mean ± standard deviation. One-way ANOVA with Tukey’s post-hoc test was used to compare multigroup data whereas repeated-measures ANOVA was utilized to compare multigroup data at different time points, followed by Tukey’s post-hoc test. *n* = 5 mice/group.

Nude mouse xenograft experiments were conducted to further verify the results of the previous experiments. Cells treated with sh-NC, sh-TET1, sh-NC + LLY-507, sh-TET1 + LLY-507, sh-NC + AZ-505, or sh-TET1 + AZ-505 were planted subcutaneously into nude mice. The results showed that the tumor size and weight of the nude mice were increased after sh-TET1 treatment while an opposite result was noted in the presence of LLY-507 or AZ-505. In addition, tumor size and weight showed a more pronounced increase upon sh-TET1 + LLY-507 or sh-TET1 + AZ-505 than LLY-507 or AZ-505 alone, respectively (Figs. 6A, S6). The tumor volume showed the same results as tumor weight (Fig. 6B). Besides, SA-β-gal staining results manifested a reduction in the number of senescent cells following TET1 silencing while the number of senescent cells was increased in LLY-507- or AZ-505-treated mice. Fewer senescent cells were observed following treatment with sh-TET1 + LLY-507 or sh-TET1 + AZ-505 than individual treatment with LLY-507 or AZ-505, respectively (Fig. 6C). Meanwhile, TUNEL staining data indicated that the number of apoptotic cells was decreased in mice treated with sh-TET1 but it was enhanced by treatment with LLY-507 or AZ-505. Conversely, the effect of LLY-507 or AZ-505 was reversed by further sh-TET1 treatment (Fig. 6D). Therefore, SMYD2 stimulated tumor growth and depressed cell senescence and apoptosis by regulating the EZH2/TET1 axis.

## Discussion

GIST is the most common type of mesenchymal carcinoma in the gastrointestinal tract, with annual incidences of 11 to 19.6 per million populations worldwide [25]. Although there is accumulating evidence revealing the molecular mechanism of GIST, most of them were focused on the abnormal activation of KIT and PDGFRA mutations [26], and the molecular mechanism underlying the pathogenesis of GIST is yet to be thoroughly investigated. Thus, exploring novel molecular targets contributing to the complete repression of tumorigenesis is vital when developing an effective therapy for GIST. The present research was designed to figure out the impact of SMYD2 on GIST. Our findings demonstrated that SMYD2 could epigenetically enhance the protein stability of EZH2 via methylation modification at the K307 position to repress TET1 through H3K27me3 modification at TET1 promoter region, resulting in a further decrease in tumor suppressor gene p53 expression and contributed to the tumorigenesis of GIST.

Specifically, gastrointestinal tissues obtained from GIST patients presented with an aberrant overexpression of EZH2, and the silencing of EZH2 in GIST cells could markedly limit the proliferation and cell cycle progression and accelerate cell senescence. In line with our findings, numerous studies have confirmed upregulated expression of EZH2 in cancers [7]. For instance, anaplastic thyroid carcinoma tissues were observed to have overexpressed EZH2 [8]. Similar findings were observed in prostate cancer tissues [27]. Also, elevated EZH2 expression was revealed in gastric cancer tissues [28]. Moreover, EZH2 is involved in various physiological or pathological processes, most of which are achieved through the regulation of cell growth, proliferation, senescence, or apoptosis [29–32]. Consistently, prior research discovered that EZH2 knockdown resulted in repression of cell proliferation and cell cycle entry in breast cancer, accompanied by a decline in cyclin D1 expression [33]. Also, EZH2 inhibition resulted in the subsequent reduction in myeloma cell proliferation and an increase in cell cycle entry [34]. Similarly, it was noted that EZH2 overexpression triggered the elevation of colon cancer cell senescence [35].

Another key finding in our study suggested that SMYD2 reinforced the stability of EZH2 protein to increase EZH2 protein expression rather than promoting EZH2 expression transcriptionally, which resulted in the stimulation of the proliferation and cell cycle entry, while inhibiting the senescence of GIST cells. According to previous reports, the modulation of SMYD2 to the EZH2 was achieved by the direct interaction of the SET domain of SMYD2 with the conserved domain II (217-329 aa, D2) of EZH2 which catalyzes the methylation of EZH2 at K307, further stabilizing EZH2 protein [10]. Furthermore, the oncogenic role of SMYD2 has been unveiled in a range of cancers. In cervical cancer, SMYD2 enhanced cell proliferation, contributing to cancer growth [21]. Consistent with our results, Kojima et al. revealed that cell viability and cell cycle were suppressed secondary to the action of SMYD2 inhibitor LLY-507 in ovarian clear cell carcinoma [36]. SMYD2 has also been found to be capable of disrupting senescence-like growth arrest that occurs as a result of RUNX upregulation in primary fibroblasts, which was in line with our results [37].

Subsequent analysis in our research provided evidence suggesting that EZH2 could repress TET1 via H3K27me3 methylation in TET1 promoter region, further downregulating p53, and that TET1 silencing resulted in the acceleration of GIST cell proliferation while suppressing cell cycle arrest and senescence. In line with our findings, prior research elucidated that EZH2 participated in H3K27me3 epigenetic regulation to downregulate TET1, subsequently blocking the antitumor p53 pathway in TNBC cells [15]. Notably, a study has elucidated that Hep-2 cell proliferation and cell cycle progression are arrested following TET1 silencing [38]. Similar to our findings, Yu et al. reported similar findings in TNBC indicating that the specific EZH2 inhibitor GSK343 or EZH2 shRNA could accelerate cell cycle arrest and senescence by upregulating TET1 expression and activating the p53 pathway [15]. Collectively, this study provided strong evidence regarding the participation of the SMYD2/EZH2/TET1 axis in GIST development and indicates that this axis could have significant potential as a therapeutic target for GIST.

However, the present study raised additional questions that require further studies. It has been identified that SMYD2 can directly methylate certain tumor suppressors like p53 and PTEN [12, 13] and that EZH2 can also target multiple other genes including RASSF1 and AXIN2 [39]. Therefore, investigation of whether SMYD2 and EZH2 can act on the tumorigenesis of GIST by regulating these molecules will be beneficial in the comprehensive understanding of the role of SMYD2 and EZH2 in the progression of GIST.

Although the SMYD2 inhibitor LLY-507 has been elucidated to repress the proliferation of esophageal, breast, and liver cancers, this compound is a potent inhibitor (<1 μM) of several enzymes (http://www.chemicalprobes.org) which could complicate the interpretation of the cell proliferation data [40]. However, it was also elaborated that SMYD2 inhibitors showed no impact on the cell proliferation of more than 240 cancer cell lines regardless of genetic or histological background. A prior study has highlighted that knockout of CRISPR/Cas9 across 313 cell lines shows no proliferative effects, and that SMYD2 is not required for autonomous proliferation of cancer cells [41]. These findings warranted further research to explore the specific mechanism of LLY-507 in GIST.

**Fig. 7:**
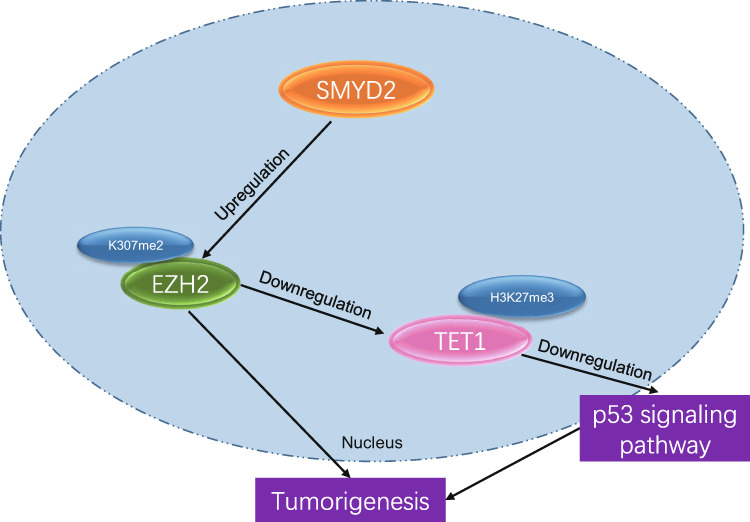
The graphical summary of the function and mechanism of SMYD2. SMYD2 elevates the expression of EZH2, promotes the methylation level of H3K27me3 in the promoter region of TET1, and reduces the expression of TET1, thus accelerating the occurrence and development of GIST.

Collectively, our findings suggest that SMYD2 could maintain the stability of EZH2 by modifying methylation at the K307 position. The stabilized EZH2 subsequently participated in the promotion of cell proliferation and suppression of cell senescence in GIST by blocking TET1 and downstream p53 signaling pathways (Fig. 7). Thereby, our results further broaden our understanding of the pathogenesis of GIST, and support the use of SMYD2 and EZH2 as potential therapeutic targets for GIST.

## Materials/subjects and methods

### Ethics statement

The experimental design was ratified by the Ethics Committee of Jingjiang People’s Hospital. The patients/participants provided their written informed consent to participate in this study. The animal study was reviewed and approved by the Animal Ethics Committee of Jingjiang People’s Hospital.

### Clinical sample collection

Tumor tissues and adjacent normal tissues were harvested from 46 patients with GIST (confirmed by pathologists and histological analysis). According to NIH criteria for tumor risk grade, the specimens were arranged into three risk groups: low, medium, and high-risk groups. Some of the collected tissues were used for RT-qPCR, some were paraffin-embedded for immunohistochemistry and the remaining was frozen in liquid nitrogen. All samples were examined by histopathologists.

### Immunohistochemistry

GIST tissue samples were prepared into paraffin sections, which were dewaxed. Following antigen retrieval with 10 mM citrate buffer (pH 6.0, heated in a pressure cooker for 2–3 min), endogenous peroxidase was blocked with 3% hydrogen peroxide solution. The sections were blocked with goat serum at 4 °C for 30 min prior to overnight incubation with rabbit antibodies (1:200, Abcam, Cambridge, UK) to EZH2 (ab186006), SMYD2 (ab234862), and TET1 (ab191698) at 4 °C. The sections were reheated for 30 min at ambient temperature, washed twice with PBS, and reacted with goat anti-rabbit IgG H&L (HRP) (1:2000, ab205718, Abcam) for 1 h. Next, the sections were developed employing DAB (P0203, Beyotime, Shanghai, China) (6 min), stained in hematoxylin (30 s), dehydrated with gradient alcohol and cleared in xylene, and sealed with neutral resin. Finally, the sections were observed under an upright microscope (BX63, Olympus Optical Co., Ltd, Tokyo, Japan). Image-Pro software was utilized to quantify the integrated optical density (OD) of the staining in the images.

### Cell culture and transfection

Human gastric mucosal cells GES-1 and human GIST cell lines GIST-T1, GIST-48, and GIST-882 were all purchased from Zeye Biotechnology Co., Ltd. (Shanghai, China) and cultured with Roswell Park Memorial Institute-1640 medium encompassing 10% FBS (Gibco, Carlsbad, CA), 10 μg/mL streptomycin, and 100 U/mL penicillin (Gibco) in a 37 °C incubator (Thermo Fisher Scientific, Waltham, MA) with 5% CO_2_.

The lentiviral packaging system was prepared based on LV5-green fluorescent protein (GFP) (lentiviral gene overexpression vector) and pSIH1-H1-copGFP (lentiviral shRNA fluorescent expression vector gene silencing vector). oe-TET1, EZH2 shRNA, and NC shRNA (sh-NC) were co-transfected into HEK293T cells using the packaged virus and the target vector. After 48 h cell incubation, the supernatant was harvested to determine the virus titer. The viruses in the exponential phase were attained and classified into three groups: sh-NC, sh-EZH2, and sh-EZH2 + OE-TET1. Next, logarithmically growing cells were treated with trypsin and titrated to the cell suspension at 5 × 10^4^ cells/mL which was then cultured in a six-well plate (2 mL/well) overnight at 37 °C. After 48 h infection, the expression of related genes was detected with the use of RT-qPCR. OE-TET1, EZH2 shRNA, and sh-NC were synthesized by GenePharma (Shanghai, China). shRNA sequences are depicted in Table S1.

In addition, cells also received treatment with dimethyl sulfoxide and LLY-507 (SMYD2 inhibitor, HY-19313, MedChemExpress, Monmouth Junction, NJ, USA) or AZ-505 (SMYD2 inhibitor, HY-15226, MedChemExpress).

### RT-qPCR

Total RNA isolated by Trizol reagent (15596026, Invitrogen, Carlsbad, CA, USA) was reversely transcribed to cDNA as per the instructions provided on the RT kit (Promega, Madison, WI, USA). RT-qPCR was implemented with a Fast SYBR Green PCR kit (Applied Biosystems, Foster City, CA, USA) on an ABI PRISM 7500 RT-PCR system (Applied Biosystems), with three replicates in each well. The relative expression of the target genes was tested by the 2^−ΔΔCt^ method and standardized by GAPDH. The primer sequences are displayed in Table S2.

### Western blot analysis

The cells collected after trypsin treatment were lysed with enhanced radio-immunoprecipitation assay lysis buffer (Boster, Wuhan, China). Following separation with 10% sodium dodecyl sulfate polyacrylamide gel electrophoresis (SDS-PAGE), protein was electro-blotted to a polyvinylidene fluoride membrane. After undergoing a 2 h membrane blocking with 5% bovine serum albumin (BSA) at ambient temperature, membrane incubation was carried out overnight with the diluted primary rabbit antibodies (1:1000, Abcam) against EZH2 (ab186006), TET1 (ab191698), cyclin A (ab181591), and p53 (ab131442) at 4 °C. The membrane was re-probed with HRP-tagged goat anti-rabbit secondary antibody (1:2000, Abcam, ab205719) at ambient temperature for 1 h before development with enhanced chemiluminescence (Millipore, Billerica, MA). The gray value of bands in western blot images was quantified utilizing the Image J software, with β-actin (ab8226, 1:1000, Abcam, mouse) as a normalizer. Each experiment was repeated 3 times.

### ChIP assay

ChIP kit (Millipore) was applied for this experiment. The 70–80% confluent cells were fixed with 1% formaldehyde at ambient temperature (10 min) to form DNA-protein crosslinking. The crosslinking was then halted by incubation with glycine solution. The cells were washed twice with cold PBS and scraped into cold PBS containing protease inhibitors. Nuclear lysate was sonicated to generate 200–300 bp DNA fragments at 120 w for 15 cycles of 2 s on and 5 s off. Subsequently, centrifugation was carried out at 4 °C and 13000 rpm. The supernatant was aliquoted into three tubes which were then supplemented with positive control antibody RNA polymerase II, NC IgG, and anti-EZH2 (1:100, Abcam, ab228697) or anti-H3K27me3 (1:100, ab6002, Abcam), respectively, for overnight incubation at 4 °C. Protein Agarose/Sepharose was introduced for the precipitation of endogenous DNA-protein complexes. The supernatant was aspirated following brief centrifugation. The non-specific complex was washed, and the precipitate was de-crosslinked at 65 °C overnight. The DNA fragment was purified and recovered by phenol/chloroform extraction. The extracted DNA and TET1 primers were used for qPCR detection. The qPCR primer sequence of TET1 is: forward: 5’-ATCTTTCCCAGAACAGCCCG-3’ and reverse: 5’-ACCATTCTACCCCCATTCTGC-3’.

### In vitro methylation and demethylation assays

Cells were incubated in methylation assay buffer (50 mM Tris-HCl, pH 8.0, 10% glycerol, 20 mM KCl, 5 mM MgCl2, 1 mM dithiothreitol, 1 mM PMSF, and 0.1 mM SAM) at 30 °C (4 h). The reaction was terminated utilizing SDS-PAGE sample buffer. Western blot analysis or mass spectrometry was implemented for evaluating the methylation status. For measuring the ability of SMYD2 to demethylate EZH2, the recombinant GST-SMYD2 (2 mg) and EZH2 K307me2 peptide were immersed in a demethylation buffer encompassing 50 mM Tris-HCl (pH 8.5), 5% glycerol, 50 mM KCl, 5 mM MgCl2, 1 mM PMSF, and 0.5% BSA at 37 °C.

### Soft agar colony formation assay

Logarithmically growing cells were suspended in the medium. Meanwhile, 1 mL of 0.5% agarose was coagulated in the 6-well plate at ambient temperature in order to prepare the bottom layer gel. Then, the upper layer gel was prepared by mixing 500 μL cell suspension encompassing 5000 cells with an equal volume of 0.5% agarose, which was covered on the bottom layer and solidified at ambient temperature. After coagulation, 2 mL medium was supplemented for 3-week incubation at 37 °C with 5% CO_2_. Following ethanol fixation and 0.1% crystal violet (C0004, Baomanbio, Shanghai, China) staining, the cells were imaged under a microscope, and the number of clones was counted with ImageJ.

### SA-β-gal assessment for cell senescence

For assessing cell senescence, SA-β-gal staining was implemented [42]. Briefly, following PBS washing, the cells were reacted with 1 ml β-gal solution (20 min), washed utilizing PBS, and reacted overnight with 1 ml β-gal solution. The cells were examined under a light microscope with an attached camera. The number of SA-β-gal-positive (blue) cells was counted, and versus the number of total cells, the percent of SA-β-gal-positive cells was calculated.

### CCK-8 assay

The cells were placed in a 96-well plate at 3000 cells/well with three parallel wells for each sample, and cultured in the incubator (37 °C, 5% CO_2_). With the medium discarded, each well of 96-well plates was added with 150 μL freshly prepared alpha minimum essential medium (α-MEM) appended to 10% CCK-8 solution (96992, Sigma-Aldrich). Meanwhile, a blank control well was prepared, which was only supplemented with a mixture of α-MEM and CCK-8. Then, the plate was incubated at 37 °C for 2 h, and the OD value was tested at 450 nm on the 1st, 2nd, 3rd, and 4th day, respectively.

### BrdU/PI staining

Cells at a density of 1 × 10^6^ cells/mL were cultured with BrdU at a final concentration of 10 mM for 30 min. The cells were suspended in 0.5 mL wash buffer and 0.5 mL of 4 M HCl and left to stand at ambient temperature for 30 min. Then, the cells were resuspended in 1 mL borax buffer and in 200 μL washing buffer before 1 h culture with 5 μL BrdU antibody at 4 °C (dark conditions). The cells were resuspended in 200 μL washing solution, followed by 30 min culture with 4 μL fluorescein isothiocyanate fluorescent secondary antibody (dark condition). After cells were suspended in 200 μL washing solution, cell culture was implemented with 200 μL PI buffer at 4 °C (15–30 min, dark condition). The cell cycle was analyzed at an excitation wavelength of 488 nm with a flow cytometer.

### Annexin V-FITC/PI double staining

Cell Apoptosis Kit (C1062S, Beyotime) was employed for cell apoptosis detection. In short, cells were centrifuged at 1000 × *g* for 5 min, whereupon the supernatant was discarded and the cells were collected, resuspended in PBS, and counted. Next, 50,000–100,000 resuspended cells were centrifuged at 1000 × *g* for 5 min, with the supernatant removed. The cells were resuspended in 195 μL Annexin V-FITC binding solution and then reacted with 5 μL Annexin V-FITC and 10 μL of PI staining solution at ambient temperature (20–25 °C) in the dark for 10–20 min. Next, the cells were subjected to an ice bath in the dark. The cells can be resuspended 2–3 times during the incubation to improve the staining effect. Fluorescence was initiated by excitation at 488 nm (FITC) and was measured by emission filters at 515 nm (FITC) and 620 nm (PI).

### Nude mouse experiment

Healthy nude mice (Beijing Institute of Pharmacology, Chinese Academy of Medical Sciences, Beijing, China, aged 6–8 weeks) were individually housed in a specific pathogen-free-level animal laboratory with humidity of 60–65% at 22–25 °C under 12 h light and dark cycle (eat and drink freely). The experiment was conducted after one week of acclimation, and the health status of the nude mice was recorded before the experiment. Differently transfected GIST-T1 cells (5 × 10^6^ cells/0.1 mL/mouse) were implanted subcutaneously in the middle and upper part of the groin, and the implanted tumor was injected with LLY-507 (1 μg/kg/day) every day (five mice for each group). Tumor formation and growth were assessed weekly, followed by calculation of tumor volume as V = ½ × a × b^2^, where a referred to length and b referred to the width of the tumors. After 4 weeks, the mice were euthanized, and the tumor size and weight were recorded. The cell senescence and apoptosis were respectively detected by SA-β-gal staining and TUNEL staining.

### TUNEL staining

The transplanted tumor tissues of nude mice were made into paraffin sections, followed by dewaxing and hydration. The removal of tissue proteins was conducted by hydrolyzing the sections with proteinase K solution (20 μg/mL) at ambient temperature for 15 min. After endogenous peroxidase had been blocked with 2% hydrogen peroxide solution, the sections were added with two drops of TdT enzyme buffer, followed by 10 min incubation at ambient temperature. Then, the TdT enzyme reaction reagent (54 μL) was added immediately into the sections for 1 h incubation in a wet box at 37 °C. The sections were positioned in a termination reaction buffer for 30 min. Two drops of peroxidase-labeled digoxin antibody were supplemented to the sections for 30 min culture in the wet box at ambient temperature. The sections were reacted with DAB solution at ambient temperature for 3–6 min before methyl green counterstaining, xylene clearing, and neutral gum mounting. The sections were observed and images were obtained under a microscope in five randomly selected fields from each section.

### Statistical analysis

All data were analyzed using SPSS 21.0 statistical software (IBM Corp. Armonk, NY, USA) with *p* < 0.05 indicating statistical significance. Measurement data were depicted as mean ± standard deviation. Paired *t*-test was processed for comparison of data between cancer tissues and adjacent normal tissues, while an unpaired *t*-test was adopted for comparison of data between the other two groups. Besides, one-way analysis of variance (ANOVA) was employed for comparison of data among multiple groups, followed by Tukey’s post-hoc test, whereas repeated-measures ANOVA was utilized for comparison of data among multiple groups at different time points, followed by Tukey’s post-hoc test. Pearson’s correlation coefficient was adopted to evaluate the relationship between two variables.

## Supplementary information

Supplementary Figures and Tables

## Data Availability

The datasets generated/analyzed during the current study are available.
