# Supplementary material for: SMYD2 aggravates gastrointestinal stromal tumor via upregulation of EZH2 and downregulation of TET1
Source: Cell Death Discov. 2022 Jun 6;8:274. doi: 10.1038/s41420-022-01038-w (PMC9170715; doi:10.1038/s41420-022-01038-w)
Supplement: Supplementary file 1 — Supplementary Figures and Tables [file 41420_2022_1038_MOESM1_ESM.docx]

**Fig. S1** **Western blot analysis manifests that EZH2 is highly expressed in GIST cell lines GIST-T1, GIST-48 and GIST-882 compared with GES-1 cell line.** * *p* < 0.05 *vs.* GES-1 cell line. Measurement data were expressed as mean ± standard deviation. One-way ANOVA with Tukey’s post hoc test was used to compare multi-group data. The cell experiment is repeated 3 times.


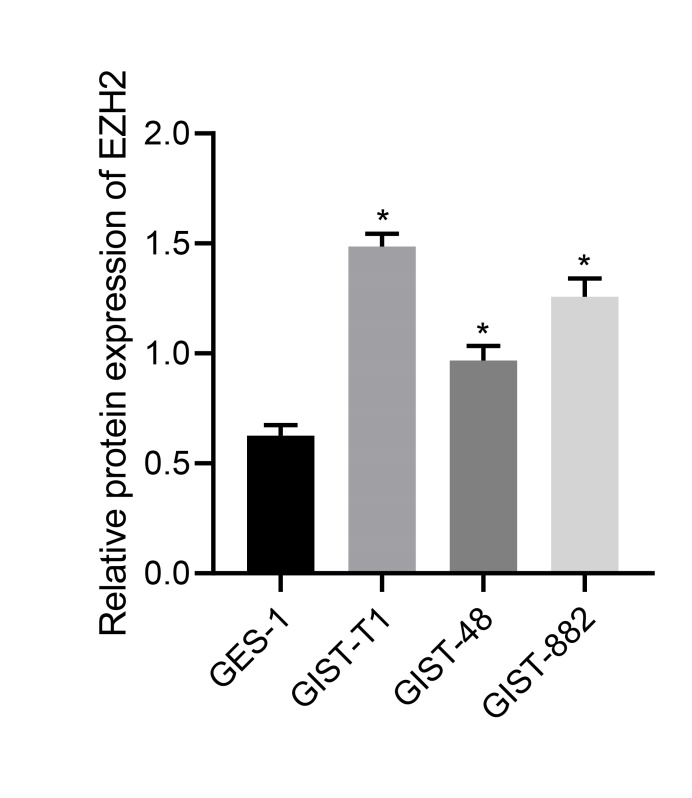


**Fig. S2 SMYD2 inhibition represses the proliferation and accelerates the senescence of GIST cells through reducing EZH2 expression.** A, RT-qPCR and Western blot analysis of EZH2 expression in GIST-T1 cells treated with AZ-505. GIST-T1 cells were treated with AZ-505 or combined with sh-EZH2. B, Western blot analysis of EZH2 protein expression in GIST-T1 cells. C, CCK-8 assay of GIST-T1 cell viability. D, GIST-T1 cell colony formation ability evaluated by soft agar colony formation assay. E, SA-β-gal detection of the proportion of senescent GIST-T1 cells. F, GIST-T1 cell cycle distribution measured by BrdU/PI cell cycle assay. G, Annexin V-FITC/PI double staining of GIST-T1 cell apoptosis. * *p* < 0.05 *vs.* control or sh-NC-, or sh-NC + AZ-505-treated GIST-T1 cells. Measurement data were expressed as mean ± standard deviation. Unpaired *t*-test was used for two-group data comparison. The cell experiment was repeated 3 times.

**
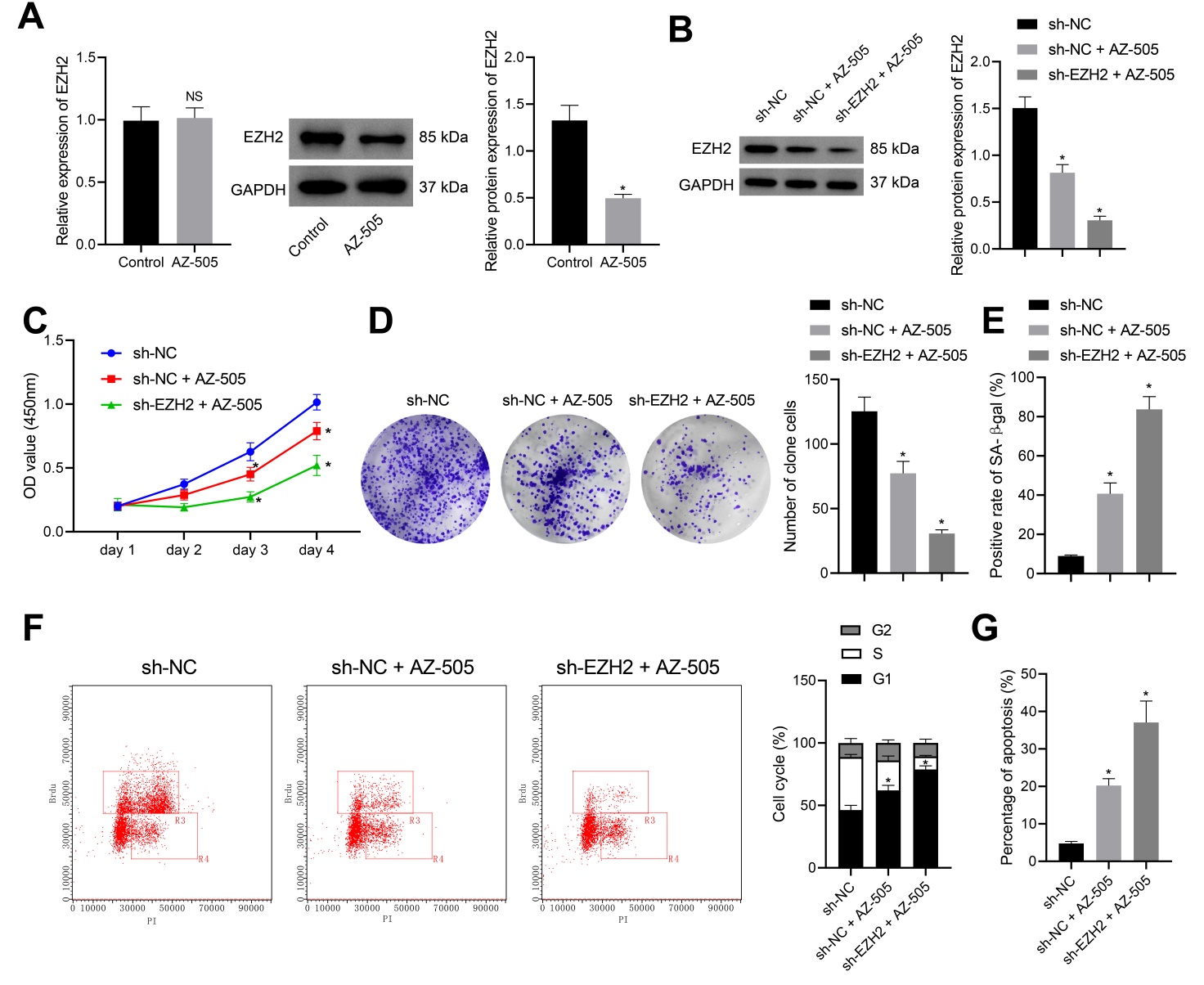
**

**Fig. S3** **SMYD2 promotes EZH2 stability through EZH2-K307 methylation.** A, Western blot analysis of EZH2 protein stability after CHX treatment. B, Western blot analysis of SMYD2, EZH2, p53 K310me2 and EZH2 K307me2 proteins in LLY-507-treated cells. C, p53 K310 methylation level in LLY-507-treated cells. * *p* < 0.05, *vs.* FLAG-EZH2 WT or control. Measurement data were expressed as mean ± standard deviation. Unpaired *t*-test was used for two-group data comparison. The cell experiment was repeated 3 times.


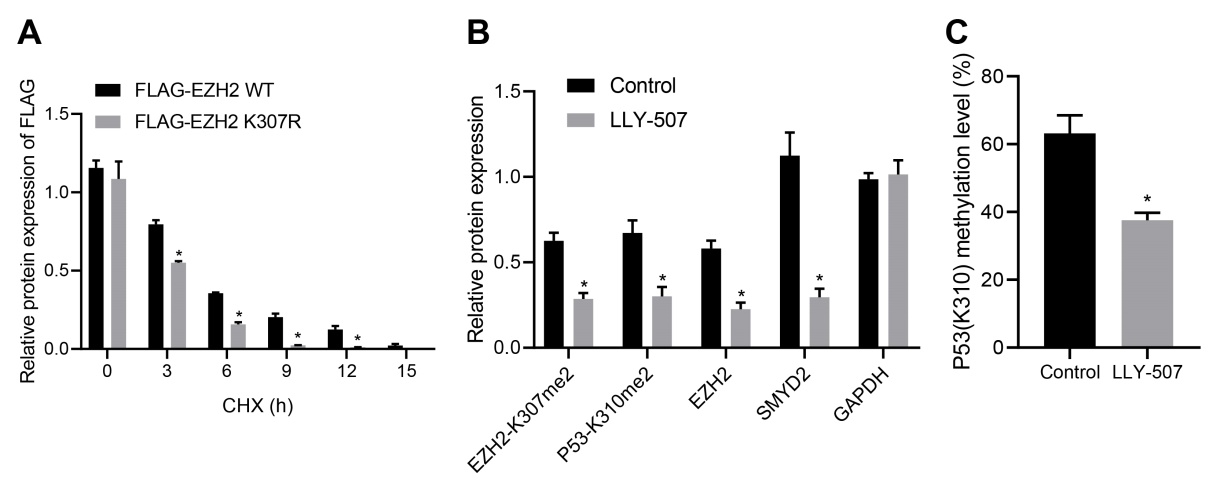


**Fig. S4 SMYD2 inhibition arrests GIST cell proliferation and stimulates cell senescence by reducing EZH2 expression.** A, RT-qPCR and Western blot analysis of EZH2 expression in GIST-T1 cells treated with sh-SMYD2. GIST-T1 cells were treated with SMYD2 KO or combined with sh-EZH2. B, Western blot analysis of EZH2 protein expression in GIST-T1 cells. C, CCK-8 assay of GIST-T1 cell viability. D, GIST-T1 cell colony formation ability evaluated by soft agar colony formation assay. E, SA-β-gal detection of the proportion of senescent GIST-T1 cells. F, GIST-T1 cell cycle distribution measured by BrdU/PI cell cycle assay. G, Annexin V-FITC/PI double staining of GIST-T1 cell apoptosis. * *p* < 0.05 *vs.* sh-NC- or sh-NC + SMYD2 KO-treated GIST-T1 cells. Measurement data were expressed as mean ± standard deviation. Unpaired *t*-test was used for two-group data comparison. The cell experiment was repeated 3 times.


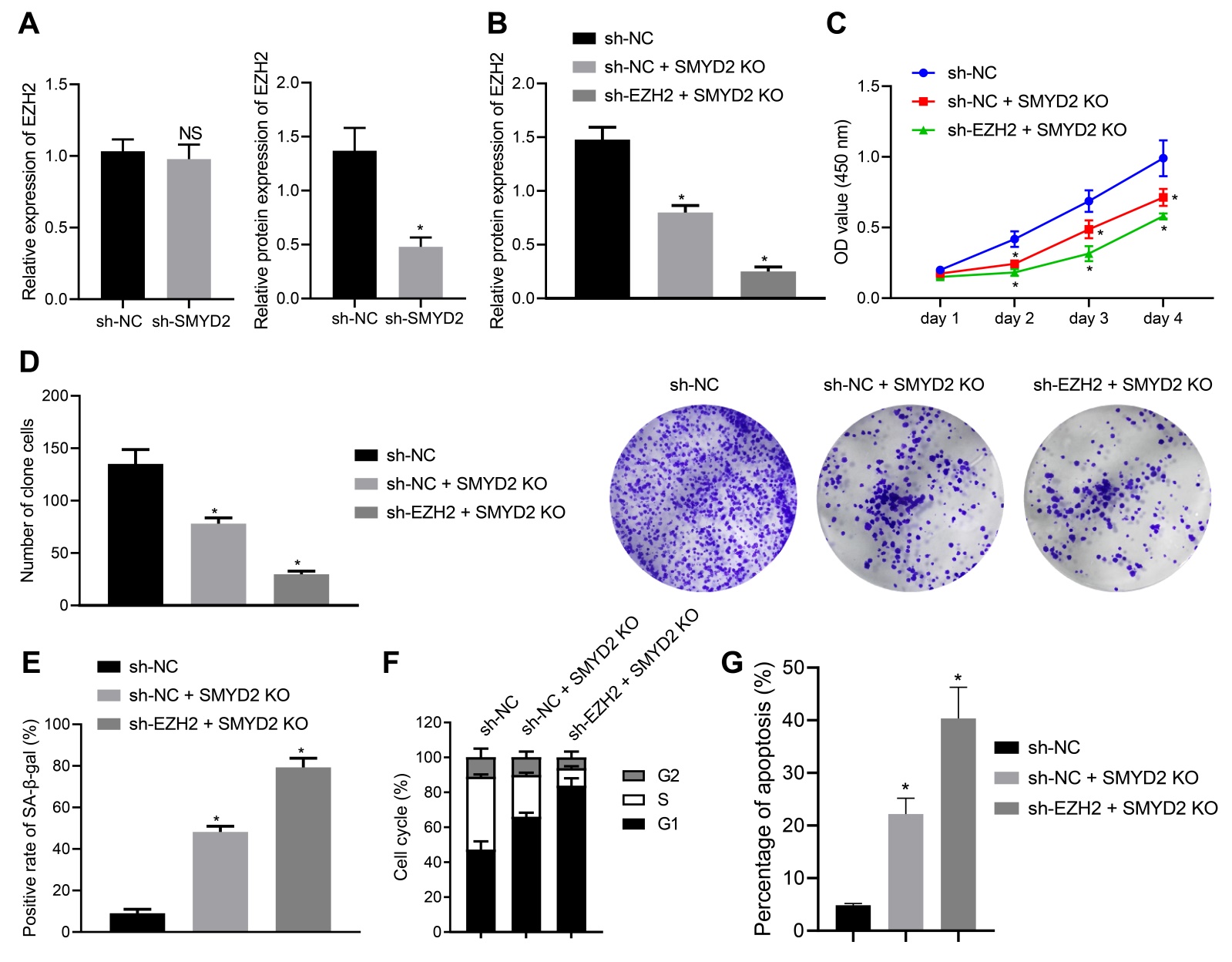


**Fig. S5** **TET1 negatively regulates TP53 pathway.** A, The enrichment of TET1 in the TP53 promoter region analyzed by ChIP experiment. B, The expression of p53, p53-S15, and p21 in sh-TET1-treated GIST-T1 cells determined by Western blot analysis. * *p* < 0.05 *vs.* sh-NC + IgG, sh-TET1 + IgG, or sh-NC. Measurement data were expressed as mean ± standard deviation. Unpaired *t*-test was used for two-group data comparison. The cell experiment was repeated 3 times.


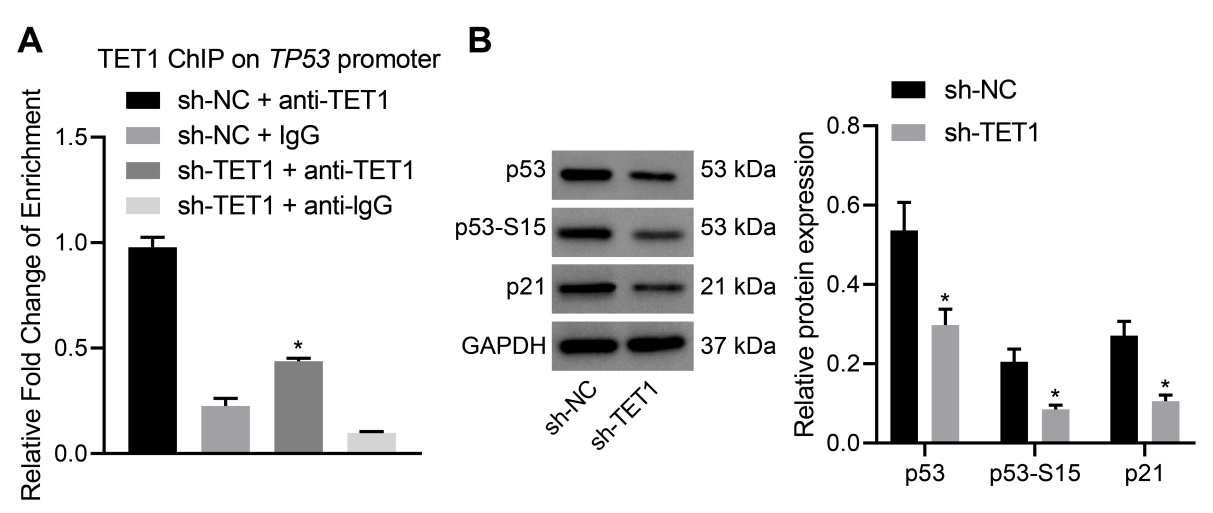


**Fig. S6** **The representative images of tumors in nude mice treated with sh-NC, sh-TET1, sh-NC + LLY-507, sh-TET1 + LLY-507, sh-NC + AZ-505, or sh-TET1 + AZ-505.**


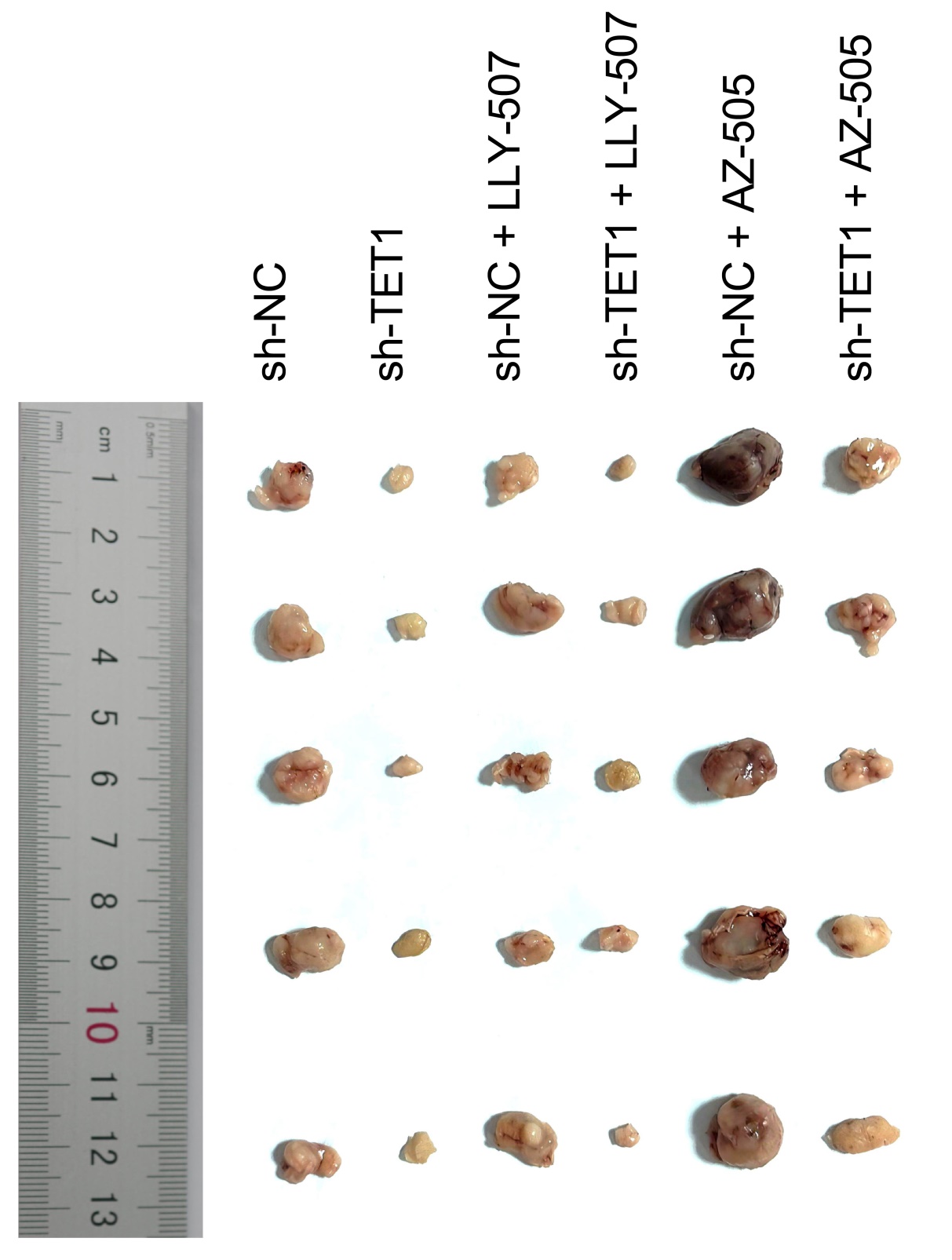


**Table S1** shRNA sequences

| shRNA | Sequence (5'-3') |
| --- | --- |
| sh-NC | CTCCAAGACUGCUCGACUAA |
| sh-EZH2 | TATGATGGTTAACGGTGATCA |

**Table S2** Primer sequences of RT-qPCR

| Genes | Primer sequence |
| --- | --- |
| EZH2 | Forward: 5’-GGACCACAGTGTTACCAGCAT-3’ |
|  | Reverse: 5’-GTGGGGTCTTTATCCGCTCAG-3’ |
| SMYD2 | Forward: 5’-TACTGCAATGTGGAGTGTCAGA-3’  Reverse: 5’-ACAGTCTCCGAGGGATTCCAG-3’ |
| TET1 | Forward: 5’-GATTGAGTGTGCCCGGCGAGAG-3’  Reverse: 5’-ATTCTTAGCTTCTTTAGCCTC-3’ |
| GAPDH | Forward: 5’-AACGGGAAGCTCACTGGC-3’ |
|  | Reverse: 5’-TGCTCAGTGTAGCCCAGGA-3’ |

RT-qPCR: reverse transcription quantitative polymerase chain reaction.
